# Establishing a Low-Resource Simulation Emergency Medicine Curriculum in Nepal

**DOI:** 10.15766/mep_2374-8265.10924

**Published:** 2020-07-15

**Authors:** Alfred Wang, Nicholas Saltarelli, Dylan Cooper, Yogendra Amatya, Darlene R. House

**Affiliations:** 1 Assistant Professor, Department of Emergency Medicine, Indiana University School of Medicine; 2 Ultrasound Fellow, Department of Emergency Medicine, Indiana University School of Medicine; 3 Director of Simulation Education, Department of Emergency Medicine, Indiana University School of Medicine; Associate Professor, Department of Emergency Medicine, Indiana University School of Medicine; 4 Lecturer, Department of General Practice and Emergency Medicine, Patan Academy of Health Sciences; 5 Assistant Professor, Department of Emergency Medicine, Indiana University School of Medicine; Lecturer, Department of General Practice and Emergency Medicine, Patan Academy of Health Sciences

**Keywords:** Global Health, Emergency Medicine, Simulation

## Abstract

**Introduction:**

High-fidelity medical simulation is widely used in emergency medicine training because it mirrors the fast-paced environment of the emergency department (ED). However, simulation is not common in emergency medicine training programs in lower-resourced countries as cost, availability of resources, and faculty experience are potential limitations. We initiated a simulation curriculum in a low-resource environment.

**Methods:**

We created a simulation lab for medical officers and students on their emergency medicine rotation at a teaching hospital in Patan, Nepal, with 48,000 ED patient visits per year. We set up a simulation lab consisting of a room with one manikin, an intubation trainer, and a projector displaying a simulation cardiac monitor. In this environment, we ran a total of eight cases over 4 simulation days. Debriefing was done at the end of each case. At the end of the curriculum, an electronic survey was delivered to the medical officers to seek improvement for future cases.

**Results:**

All eight cases were well received, and learners appreciated the safe learning space and teamwork. Of note, the first simulation case that was run (the airway lab) was more difficult for learners due to lack of experience. Survey feedback included improving the debriefing content and adding further procedural skills training.

**Discussion:**

Simulation is a valuable experience for learners in any environment. Although resources may be limited abroad, a sustainable simulation lab can be constructed and potentially play a supportive role in developing an emergency medicine curriculum.

## Educational Objectives

By the end of this activity, participants will be able to:

1. Demonstrate competency in caring for common emergency medicine case presentations.
2. Demonstrate specific knowledge regarding common emergency medicine cases.
3. Demonstrate communication and leadership skills and provide feedback working as a team.

## Introduction

The United States Institute of Medicine has identified the emergency department (ED) as having the hospital's highest risk for adverse events, likely due to high patient volume, acuity, and intense time pressures. In its 200-page report, the Institute of Medicine went on to recommend simulation training as an effective strategy to improve patient safety.^1^ This recommendation is supported by a substantial body of research indicating that simulation is a highly effective modality for developing medical knowledge and procedural, team, and communications skills, as well as for addressing systems-based practice challenges in emergency medicine.^2-4^ Accordingly, both the Accreditation Council for Graduate Medical Education and the Council of Emergency Medicine Residency Directors include simulation in residency training curricula as an important tool to improve and evaluate communication skills, medical knowledge, and other core competencies crucial to good patient outcomes.^5,6^ This is in stark contrast to emergency medicine training programs in developing countries, where simulation is not commonly integrated into training programs.^7^ There are multiple potential limiting factors, including cost, availability of resources, faculty experience, and buy-in from stakeholders.^8^ The current literature on low-resource simulation is mostly based on specific procedural skills such as C-sections,^9^ trauma resuscitation,^10,11^ and surgical skills.^12^ Among the 322 emergency medicine simulations published in *MedEdPORTAL,* none report on implementation in the low-resource environment of an underdeveloped nation. Authors have elsewhere reported that short courses on trauma^10,11^ and emergency care^13^ in underdeveloped settings have incorporated simulation components, without giving the specifics of their design. To our knowledge, the current publication is the first to describe the detailed particulars of the development, implementation, and assessment of a low-cost dedicated emergency medicine simulation training program in a resource-limited setting. It is our hope that this work will facilitate other efforts in the development of local simulation programs despite the significant resource limitations that are present in much of our world.

## Methods

The simulation lab was set up at the major teaching hospital in Patan, Nepal, with approximately 48,000 ED patient visits per year. This site was chosen because Indiana University had a memorandum of understanding with the hospital and regularly sent emergency medicine residents there as part of a global health elective. There was also a permanent Indiana University faculty working at the Patan Hospital ED department to facilitate cooperation among the visiting residents and the local staff.

The hospital had an emergency medicine rotation for medical students and an 18-month emergency medicine fellowship that could be completed after a general practitioner residency. Medical officers worked for 1 year in the ED after a 6-year medical school. Once they had completed their year as a medical officer, they could apply for postgraduate training. General practice residency was 3 years. Emergency medicine fellowship training was 18 months following general practice training.

Medical officers were the primary caregivers in the ED. Supervision was provided in 24-hour shifts by a staff physician who was available for optional assistance and to oversee care; however, medical officers maintained the primary responsibility of patient care and disposition.

Of a total of 35 medical officers, only 20 were available to participate in the simulation setup during their nonmandatory Tuesday didactics. Both the medical student and medical officer curriculum contained no simulation training prior to our training session.

### Equipment

The setting was a multiuse conference room in the hospital with a projector and six large tables. The simulation equipment available included one full-sized cardiopulmonary resuscitation (CPR) manikin (Resusci Anne, Laerdal Medical), and one intubation trainer (Ambu). The same equipment available in the ED was also made available in the simulation cases. This included a desktop computer and projector to simulate the patient cardiac monitor,^14^ as well as a bag valve mask, different-sized endotracheal tubes with stylets, oral airways, one bougie, and one curved laryngoscope with size 3 and 4 blades. The Table lists the items and where each was acquired. None of the items were purchased as all were available at Patan Hospital.

**Table.**
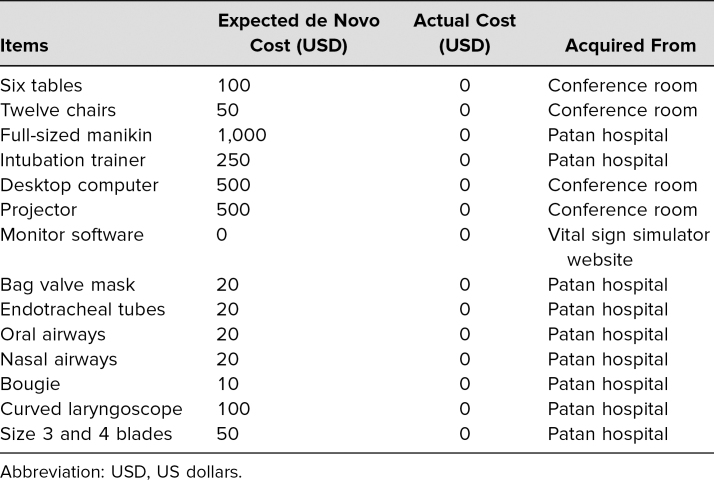
Details of Items Used for Simulation

### Simulation Case Development

An informal needs assessment was performed for case development. The monthly census of patients was reviewed, and after discussion with the local hospital ED staff, cases were developed to cover the most common scenarios seen in the ED. One procedure lab was developed per request of ED staff to improve learners' airway skills. In addition, cases were purposefully kept uncomplicated as learners did not have simulation or role-playing experience. Appendices A-H contain the simulation cases developed.

### Personnel/Implementation

One local Nepali ED faculty who received simulation training during his Nepal emergency medicine fellowship at a local private Patan hospital simulation center kept track of the simulations and was responsible for the debriefings. One American emergency medicine physician, who was faculty at Patan Hospital, served as a resource for simulation training. One American emergency medicine resident helped develop cases and assisted in running them. The learners included 35 medical students and medical officers who were working in the ED that month.

A free-to-use cardiac monitor^14^ was downloaded onto an available computer and displayed on the projector. The cardiac monitor showed active changes in blood pressure, respiratory rate, heart rate, pulse oximetry, and variable rhythms on telemetry. The program simulated defibrillation/cardioversion and pacing and was able to track events, such as when an IV was started or how long CPR was performed, for later review. During cases, there were three facilitators: One acted as the nurse; one ran the monitor, provided verbal lab results, and displayed radiographic images; and one served as the patient or family member.

Every attempt was made to simulate the resources available in the ED. Any workup such as labs or radiology was provided only if also available in the ED. These workups were also provided in a fashion that would be like those provided in the actual emergency room. Specifically, labs or radiology ordered in simulation had a delay prior to results being given as there was no point of care or portable radiology available in the actual department. Because all labs needed to be delivered to the main laboratory prior to getting results and radiology needed to have the patient go next door to the radiology department, these results were made available only after a reasonable 3- to 5-minute delay in the simulation.

IV fluids and ED medications were also available; learners were required to verbalize specific dosing. Furthermore, all dispositions available in the Patan Hospital ED (such as the ICU) were also available in the simulations.

As the manikin only allowed for CPR, it was mostly used for direct patient contact (e.g., the learners would talk directly to the manikin and perform physical exam maneuvers such as auscultation). However, for cases when the simulation involved a cardiac arrest, the manikin allowed for direct CPR.

Simulation cases were given during nonmandatory biweekly 2-hour slots intended for lectures for medical officers and students. Learners were broken up into teams of four, with a medical officer serving as team leader and assigning roles prior to the case starting. If medical students were participating, they served as team members. Due to space and equipment limitations (only one monitor), cases were run one at a time.

Cases included trauma with tension pneumothorax, ST elevation myocardial infarction complicated with ventricular fibrillation, pneumonia with septic shock, organophosphate poisoning, Advanced Cardiovascular Life Support (ACLS) cardiac arrest, anaphylaxis, and trauma with large subdural hematoma (see Appendices A-H). Cases typically lasted 20 minutes, with an additional 30 minutes for debriefing.

One procedure lab was done as well for airway management. It incorporated techniques such as bag valve mask and bougie-assisted intubation (see Appendix H).

All debriefings were led by the Nepali ED faculty with previous simulation training. Debriefing was done in the same room after each case, which allowed trainees, facilitators, and observers to comment on the scenario. The crucial topics discussed included feedback on teamwork and communication and review of medical management.

### Assessment

After the end of the entire series (a total of 4 simulation days), learners were given a voluntary electronic survey with two open-ended questions: “What did you enjoy from simulation?” and “What improvements can be implemented to enhance your learning?” (Appendix I). Though not piloted, this scaled-down evaluation approach was done in order to allow participants new to simulation to guide the development of future cases.

A thematic analysis was conducted on the results of the survey by the primary author in conjunction with the Nepali ED faculty involved.

## Results

Eight cases were run over 4 separate days. Thirty-five learners (including 15 medical students and 20 medical officers) participated. On average, each learner participated in two cases. Of the 20 medical officers who participated in simulation, 10 completed the optional electronic survey. Medical students were excluded from the survey as they mostly played an observational role during the simulation cases.

A thematic analysis of participant comments from the survey was conducted. The most-common answer for what learners enjoyed was working as a group, while the most-commented area to improve upon was debriefing content. Representative quotations from participants for each theme are shown below:

• What did you enjoy?
  ○ “Working as a group.”
  ○ “Being more confident for live cases.”
  ○ “Safe learning space.”
  ○ “Better retention than book learning.”
• What can be improved?
  ○ “Further re-cap of the medical point of cases.”
  ○ “Participation of all team members besides team leader.”
  ○ “Want further cases in pediatric and or ob/gyn patients.”
  ○ “Specific procedure skills such as chest tube, central line, cricothyrotomy.”

## Discussion

We successfully developed and implemented a novel, low-fidelity simulation for house officers and students in a resource-limited setting. Simulation sessions were well received by the learners in our study.

One of our goals was stressing the importance of communication. In the ED, house officers often do not have to vocalize many orders in a critical case as nurses would perform activities without being asked, but in simulation, they had to learn to vocalize what they wanted and assign roles in order to provide the appropriate care for the patient. For example, in our intubation procedural lab, house officers worked on leading an intubation: calling out for suction, preoxygenating, and selecting appropriate airway equipment and medications. Our survey results parallel those from a simulation-based emergency team coordination course by Shapiro and colleagues^15^: Simulation training led to improved communication and teamwork.

We also aimed to improve patient care through increasing knowledge and comfort related to commonly faced scenarios in the Patan Hospital ED. Learners reported that they appreciated the practical teaching of medical concepts in a safe environment. Similar to outcomes studied by Mueller and colleagues, where medical students were trained in common ACLS cases, our participants believed simulation was effective in linking medical theory to actual practice and felt more confident with handling real-life cases thereafter.^16^

One of our initial challenges was preparing learners for simulation and adapting simulation to accommodate Nepalese learners. The initial case proved most difficult because learners had never had simulation as part of their medical training. As a result, they were very hesitant to act out the scenario and needed direction. Medical officers commented that they had difficulty leading a simulated intubation because they were not sure what was expected in the simulation lab. To address this, we discussed solutions with the Patan Hospital staff who had been trained at the hospital. After collaboration, we explicitly set expectations regarding roles, verbalizing orders, and closed-end communication in a presimulation orientation moving forward. We also emphasized the importance of treating each simulation as a real-patient encounter.

Over the course of the subsequent simulation cases, we observed an improvement in learners' communication skills and comfort with simulation. Initially, learners often looked to the facilitator for assistance, but over the course of simulation, they began running cases independently. Expectations were revisited after the initial airway simulation and debriefings became more involved: Learners asked more questions regarding management and demonstrated more detailed self-assessment on self-improvement.

Despite learners' increased comfort, we often had to use the nurse role to prompt them for key areas of management, such as change in vital signs, abnormal heart tracing, and availability of airway backup during intubation. This differed from the actual nursing role in the Patan Hospital ED, where most nurses would perform the next step without being asked (e.g., placing pads on, gathering all the airway supplies including a bougie for backup). In the simulations, we wanted learners to think of critical steps themselves; therefore, the facilitator in the nursing role did not perform steps independently. This was particularly important as the learners might eventually practice in an environment where nurses were not able to anticipate needs, requiring development of communication skills and anticipation of steps in care.

Another challenge we encountered was that learners requested more time on debriefing. Initially, we spent equal time on the case and debriefing because learners were new to simulation/role-playing and needed more guidance during a simulation. After one to two simulation cases, the learners became more confident, and we were able to lengthen the debriefing time for subsequent simulation cases. However, we hope to continue to improve and encourage further education with potential prereadings and further in-depth discussions during debriefings.

Despite our success, the generalizability of our setup for resource-limited settings may be challenging as local resources may be different. There were no costs in developing our simulation lab since we were able to use locally available resources. The only resource that we contributed was the free-to-use monitor software.

Regarding generalizability of the simulation content, Patan Hospital included most specialists (except neurosurgery, neurology, and interventional cardiology), adult and pediatric intensive care (with no ventilator available in the ED), six cardiac monitors, full laboratory services, chest X-ray, electrocardiogram, ultrasound, and kits for common procedures such as chest tubes and central lines. Services like computed tomography and magnetic resonance imaging, however, were less readily available and were not offered to learners during simulation. Resource allotment will likely vary in different lower-resourced hospital settings.

Another challenge to generalizability is availability of a local provider who has simulation training. We were able to set up a long-term sustainable simulation curriculum because the provider was already comfortable with simulation and debriefing. Furthermore, he trained at Patan Hospital and was able to provide feedback and changes in order to accommodate the local Nepalese learners. Since the development of the initial cases, he and the American emergency medicine physician, who is faculty at Patan Hospital, have continued to develop simulation cases for learners during the emergency medicine rotation. We plan to continue this work with him for ongoing training and case development in order to maintain this curriculum's success.

Another limitation of our study was our simplistic evaluation method and the limited responses we received (10 medical officers out of a potential 20 responded). Because simulation was completely novel in the curriculum, we chose the simplistic method of evaluation but did not make it mandatory, which likely contributed to our limited responses. We included only two questions in our survey to encourage simple open-ended answers: for the learners to easily communicate what they wanted to change and for the staff to address one or two potential improvements without becoming too complicated. In future evaluations, we aim to include self-assessments as well as learner perception of how low-fidelity simulation relates to real-life resuscitations and patient care.

Besides improving our future evaluations, we also plan to involve the nursing staff in an effort to improve communication between them and residents in the Patan Hospital ED and to encourage interdisciplinary education and debriefing. We also plan to create more procedural simulations as well as incorporating more pediatric and obstetric cases, although, with only a unisex adult simulation model, these cases will require more creativity. Finally, we aim to further improve our debriefing in response to learners' requests for more-detailed teaching focused on pathophysiology, etiology, diagnosis, and management of the specific conditions featured in the simulation cases. In summary, simulation is a valuable experience for learners in any environment. Although resources may be limited abroad, a simulation lab can still be possible and sustainable and play an important role in any developing emergency medicine curriculum.

## Appendices

Trauma With Tension Pneumothorax.docxMyocardial Infarction With V-fib.docxPneumonia With Septic Shock.docxOrganophosphate Poisoning.docxACLS Cardiac Arrest.docxAnaphylaxis.docxTrauma With Subdural Hematoma.docxProcedure-Specific Lab.docxSimulation Curriculum Survey.docx
*All appendices are peer reviewed as integral parts of the Original Publication.*
