# Supplementary material for: Establishing a Low-Resource Simulation Emergency Medicine Curriculum in Nepal
Source: MedEdPORTAL. 2020 Jul 15;16:10924. doi: 10.15766/mep_2374-8265.10924 (PMC7373349; doi:10.15766/mep_2374-8265.10924)
Supplement: Supplementary file 1 — Trauma With Tension Pneumothorax.docxMyocardial Infarction With V-fib.docxPneumonia With Septic Shock.docxOrganophosphate Poisoning.docxACLS Cardiac Arrest.docxAnaphylaxis.docxTrauma With Subdural Hematoma.docxProcedure-Specific Lab.docxSimulation Curriculum Survey.docx [file mep_2374-8265.10924-s001.zip › A. Trauma With Tension Pneumothorax.docx]

| **Appendix A: Motor vehicle accident with tension pneumothorax**  **SIMULATION CASE TITLE: Motor vehicle accident (MVA) with tension pneumothorax**  **AUTHORS: Alfred Wang MD** | |
| --- | --- |
| **PATIENT NAME: Newar**  **PATIENT AGE: 44 years old**  **CHIEF COMPLAINT: chest pain, shortness of breath** | |
|  | |
| ***Brief narrative description of case*** | *Patient presents after a single vehicle MVA at highway speeds complaining of chest pain and shortness of breath. Learners should be able to perform a primary survey and diagnose/manage a tension pneumothorax.* |
| **Primary Learning Objectives** | 1. *Demonstrate performance of primary and secondary survey and organize a trauma resuscitation* 2. *Demonstrate ability to formulate a differential diagnoses of post traumatic shortness of breath* 3. *Describe the management of tension pneumothorax* |
| **Critical Actions** | 1. *The learner will take lead and assign clear roles*  - *Ask for help* - *Ask for intravenous (IV) access, to have patient placed on monitor, call for nasal cannula* - *Ask for vital signs*  1. *The learner will perform a primary survey*  - *Assess airway, breathing and circulation*  1. *The learner will intervene appropriately for the tension pneumothorax*  - *If learner chooses to do needle decompression, describe the procedure:*   - *Prepare equipment: personal protective equipment (PPE), 18 gauge needle, cleaning solution*   - *Describe procedure of needle decompression:*     - *Describe anatomy: midclavicular line over 2^nd^ intercostal space*     - *Sterilize field and place on personal protective equipment*     - *Insert large bore needle at 90^o^ angle to chest wall until hear a “pop” and a rush of air through needle*     - *Ask for confirmatory imaging* - *If learner chooses to do chest tube, describe the procedure*   - *Prepare equipment: PPE (a white coat and gloves), scalpel, cleaning solution, drape, analgesia, chest tube, suture kit, sutures, curved Kelly clamp, closed drainage system, gauze for dressings*   - *Describe procedure of chest tube insertion:*     - *Identify insertion site: 4^th^ or 5^th^ intercostal space in mid-to-anterior axillary line.*     - *Position patient with arm on affected side over patient’s head*     - *Prep the patient’s skin with cleaning solution*     - *Drape patient*     - *Administer analgesia*     - *Make 2-4 cm transverse incision through skin and subcutaneous tissues. Dissect down to pleura. Placing a hemostat (no Kelly clamps available in Patan’s chest tube kit) above the rib, push through pleura and expand space prior to withdrawing.*     - *Insert index finger to ensure in correct space*     - *Place chest tube and direct in direction of patient’s head and anteriorly*     - *Connect chest tube to closed suction system*     - *Suture down chest tube*     - *Dress chest tube with gauze*     - *Ask for confirmatory imaging*  1. *The learner will communicate effectively with other members of the hospital system and disposition the patient appropriately*    - *Learner will call general surgery*    - *Describe case succinctly and arrange for proper disposition* |
| **Learner Preparation** | *There is no pre-reading.*  *Learners will be told that the patient is a 44 year old male that weighs around 70 kilograms. He was involved in a single vehicle MVA at highway speeds and was brought in by bystanders. He is complaining of chest pain and shortness of breath.* |

| Initial Presentation | | | |
| --- | --- | --- | --- |
| **Initial vital signs** | HR 140, BP 75/35, RR 26, T: 36.2, O2 sat (room air) 75% | | |
| **Overall Appearance** | *Patient in distress and diaphoretic. Clutching chest.* | | |
| **Actors and roles in the room at case start** | *There is an actor playing a nurse in the room. She is a new nurse and asks for direction.* | | |
| **HPI** | *Please specify what info here and below must be asked vs. what is volunteered by patient or other participants.*  *Information provided by patient:*  *44 year old with no medical conditions.*  *No allergies.*  *Not on medications.*  *Otherwise patient will not answer other questions: just screaming in pain and in distress.* | | |
| **Past Medical/Surgical History** | **Medications** | **Allergies** | **Family History** |
| None | None | None | Non-contributary |
| **Physical Examination** | | | |
| **General** | Well-nourished man in severe distress, clutching chest and screaming in pain | | |
| **HEENT** | No signs of head trauma. No drainage from nose. | | |
| **Neck** | Trachea deviated toward L side | | |
| **Lungs** | No lung sounds on R side | | |
| **Cardiovascular** | Sinus tachycardia, cap refill> 4 seconds, weak distal pulses. | | |
| **Abdomen** | Soft, non-tender, no signs of bruising | | |
| **Neurological** | AVPU: V classification. Answers some questions but otherwise in too much distress to answer anything else. Moving all four extremities spontaneously. Pupils are equal and reactive. | | |
| **Skin** | Normal | | |
| **GU** | Normal | | |
| **Psychiatric** | Distressed | | |

| Instructor Notes - Changes and CASE Branch Points | | |
| --- | --- | --- |
| **Intervention / Time point** | **Change in Case** | **Additional Information** |
| *Learner requests CXR* | *Learner shown CXR in multimedia demonstrating pneumothorax after a delay of 3-5 minutes, in which time the patient will get sicker- BP decreases to 60/30 and sats will decrease in the 60s.* | *CXR is available in Patan hospital but patient will have to be wheeled to get CXR.* |
| *Learner requests EKG* | *Learner shown EKG in multimedia* | *EKG is available immediately at Patan emergency room so is readily available in simulation.* |
| *Learner decides to intubate instead of addressing pneumothorax* | *Vitals do not improve. BP decreases to 60/30 and sats remain in the 70s.* | *RN asks “doctor, are we missing something?”* |
| *2-3 minutes into case- lower BP and decreasing saturations* | *BP begins decreasing if tension pneumothorax is not recognized*  *O2 sat decreases if not placed on oxygen source* | *RN tells the provider that the BP is now in the 60s and now saturations in 60s* |
| *No analgesia given prior to chest tube placement* | *Patient screams loudly in pain* |  |
| *After chest tube placement* | *Patient is not in distress anymore and BP/O2 sat normalizes to 140/90 and 99% respectively* | *Patient states: “I feel much better”* |
| *Learner requests post-chest tube CXR* | *CXR machine down currently.* |  |

**Ideal Scenario Flow**

*The learners walk to the patient to find him in severe distress. The leader asks the nurse to immediately place the patient on the monitor; calls for IV access and supplemental oxygen and vitals. Leader will perform a primary survey and recognize that airway is intact. He/she will recognize that on breathing, the patient has a tension pneumothorax. He/she will treat the tension pneumothorax appropriately. Once chest tube/or needle decompression is performed, patient will improve and the learner should finish the primary survey. The learner should call general surgery for ultimate disposition. If obtained, chest x-ray will show a tension pneumothorax (pre-chest tube) and will show a correctly placed chest tube (post-chest tube). If obtained, EKG will demonstrate sinus tachycardia. If asked, no laboratory studies will return during the case.*

**Anticipated Management Mistakes**

1. *Delay in management of tension pneumothorax*: *Learners diagnosed tension pneumothorax readily but were more reluctant to manage. This is likely due to lack of experience. We found it helpful to allow the nurse to prompt for the next step and the patient vitals to decompensate further if prompt chest tube placement was not performed.*
2. *Failure to lead team:* *Our learners in Nepal tended to be very quiet and needed prompting. The nurse had to ask for help multiple times to get direction. Learners became very focused on making a diagnosis and sometimes forgot direct the rest of the team.*

Multimedia


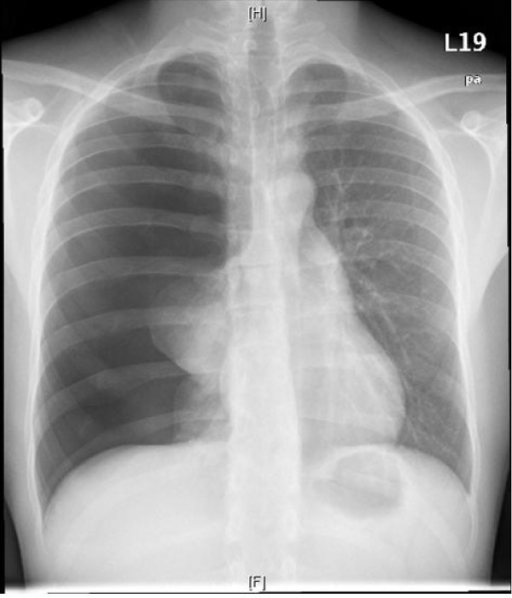


Image by [Krankenhaus München Harlaching], retrieved from: [https://openi.nlm.nih.gov/detailedresult.php?img=PMC3177884_1749-7922-6-32-1&req=4] on [12/07/18]. Creative Commons License associated: [https://creativecommons.org/licenses/by/2.0/]


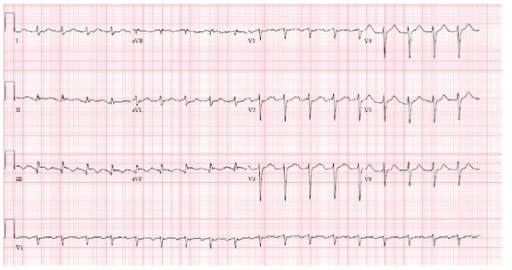


Image by [Department of Internal Medicine, University of Floria College of Medicine], retrieved from: [https://openi.nlm.nih.gov/detailedresult.php?img=PMC4055418_CRIC2014-120607.001&req=4] on [12/07/18]. Creative Commons License associated: [https://creativecommons.org/licenses/by/3.0/]
