# Supplementary material for: Establishing a Low-Resource Simulation Emergency Medicine Curriculum in Nepal
Source: MedEdPORTAL. 2020 Jul 15;16:10924. doi: 10.15766/mep_2374-8265.10924 (PMC7373349; doi:10.15766/mep_2374-8265.10924)
Supplement: Supplementary file 1 — Trauma With Tension Pneumothorax.docxMyocardial Infarction With V-fib.docxPneumonia With Septic Shock.docxOrganophosphate Poisoning.docxACLS Cardiac Arrest.docxAnaphylaxis.docxTrauma With Subdural Hematoma.docxProcedure-Specific Lab.docxSimulation Curriculum Survey.docx [file mep_2374-8265.10924-s001.zip › B. Myocardial Infarction With V-fib.docx]

| **Appendix B: ST elevation myocardial infarction (STEMI) with ventricular fibrillation cardiac arrest**  **SIMULATION CASE TITLE: ST elevation myocardial infarction (STEMI) with ventricular fibrillation cardiac arrest**  **AUTHORS: Alfred Wang MD** | |
| --- | --- |
| **PATIENT NAME: Milan**  **PATIENT AGE: 60 years old**  **CHIEF COMPLAINT: Chest pain** | |
|  | |
| **Brief narrative description of case** | *60 year old male coming from home with chest pain for 2-3 hours.*  *Learners are expected to recognize STEMI on EKG and perform appropriate diagnostic workup. Also learners are expected to recognize ventricular fibrillation cardiac arrest (Vfib arrest) and lead a resuscitation.* |
| **Primary Learning Objectives** | 1. *Demonstrate the ability to organize and lead the care team.* 2. *Apply history and physical gathering skills to correctly order an early EKG to diagnose and treat a STEMI.* 3. *Distinguish cause of arrest as Vfib arrest and apply ACLS (Advance Cardiac Life Support) skills to resuscitate patient* 4. *Recognize catheterization lab as final disposition for patient.* |
| **Critical Actions** | 1. *The learner will take lead and assign clear roles*  - *Ask for help* - *Ask for intravenous (IV) access, to have patient placed on monitor, call for nasal cannula* - *Ask for vital signs*  1. *Promptly perform focused History and Physical Exam*  - *Discover complaints of chest pain and diaphoresis and radiating pain*  1. *Promptly obtain EKG*  - *Recognize ST elevations in leads V2-V6, I and aVL and reciprocal ST depression in leads II, III, aVF*  1. *Give 325 mg of oral aspirin and consider medications such as nitroglycerin after confirming normal blood pressure* 2. *Order appropriate labs: troponin, CBC, BMP, coagulation panel* 3. *Intervene when patient goes unresponsive*  - *Feel for pulse and ask for cardiopulmonary resuscitation (CPR) to commence upon discovering no pulse* - *Look at monitor and correctly diagnose Vfib arrest* - *Ask for defibrillator and defibrillate patient* - *Continue CPR with pulse and rhythm checks every 2 minutes* - *Give 1mg of (1:10,000) IV epinephrine every 3-5 minutes*  1. *Once patient has return of spontaneous circulation, stop CPR* 2. *The learner will communicate effectively with other members of the hospital system and disposition the patient appropriately*  - *Call cardiology* - *Describe case succinctly and send patient to catheterization lab*  1. *Provide effective team leadership*  - *Verbally assign roles* - *Provide specific instructions* - *Remain calm* |
| **Learner Preparation** | *There is no pre-reading.*  *60 year old male coming in from home with chest pain that started 3 hours prior to arrival.* |

| Initial Presentation | | | |
| --- | --- | --- | --- |
| **Initial vital signs** | HR 80, BP 110/60, RR 14, Temp 37, Room air O2 saturation 99% | | |
| **Overall Appearance** | *Patient diaphoretic and dry heaving.* | | |
| **Actors and roles in the room at case start** | *Nurse in room.*  *Family member (optional) can present history and demonstrate concern.* | | |
| **HPI** | *Patient volunteers history of present illness: says chest pain has been going on for 3 hours. Was working in house when he felt the pain. Only getting worse. Pain is crushing and radiates down L arm. He says he has never felt like this before. Endorses nausea.*  *If asked about any leg swelling, recent travel, hemoptysis, history of blood clots, patient will deny.*  *Review of systems is otherwise negative.* | | |
| **Past Medical/Surgical History** | **Medications** | **Allergies** | **Family History** |
| Hypertension  Diabetes | Lisinopril  Metformin | None | Non- contributory |
| **Physical Examination** | | | |
| **General** | Patient uncomfortable and diaphoretic | | |
| **HEENT** | Diaphoretic. Otherwise normal. | | |
| **Neck** | Soft, supple. Normal | | |
| **Lungs** | Clear lungs bilaterally | | |
| **Cardiovascular** | Normal sinus rhythm. Regular rate. Cap refill < 3 seconds. No murmurs. Brisk distal pulses. | | |
| **Abdomen** | Soft, non-distended. | | |
| **Neurological** | Awake, alert, oriented x 3. No focal neurologic deficits. | | |
| **Skin** | Normal | | |
| **GU** | Normal | | |
| **Psychiatric** | Normal affect | | |

| Instructor Notes - Changes and CASE Branch Points | | |
| --- | --- | --- |
| **Intervention / Time point** | **Change in Case** | **Additional Information** |
| *3 minutes into the case* | *Patient becomes unresponsive. Monitor will demonstrate Vfib.*  Patient will have no pulses. | *RN will ask the provider what to do.* |
| *Team asks for EKG* | *EKG in multimedia shown to learners* |  |
| *Team asks for CXR* | *CXR in multimedia shown to learners after a delay of 3-5 minutes.* | *CXR is available in Patan hospital but patient will have to be wheeled to get CXR.* |
| *If not recognize ventricular fibrillation* |  | *RN will ask provider why the rhythm strip on the monitor “looks funny”* |
| *2 rounds of CPR is given (2 minutes each) and one round of epinephrine given* | *Pulses will be present.*  *Vitals will be BP 180/90, HR of 120, O2 sat on RA 99%, RR 18.*  *Patient will become alert and oriented.* | Patient will ask “what just happened?” |
| *Call another service than cardiology for disposition* |  | *Physician on that service will be angry and ask why there are calling them. Will ask if they called cardiology.* |
| *Did not give aspirin* |  | *Cardiology will ask why the patient never got aspirin when called.* |

**Ideal Scenario Flow**

*The learners enter the room to find the patient diaphoretic and complaining of active chest pain. They ask the nurse to patient on the monitor, obtain IV access, and ask for a set of vitals. When they ask for an EKG, person running the simulation should pull up an example of an EKG with an anterior STEMI and show it to learners. Learners recognize that is a STEMI and order labs, including troponin. They also order aspirin for the patient. Five minutes into the case patient goes unresponsive. Learners recognize Vfib on the monitor and direct the team to start CPR per ACLS protocol. After two rounds of CPR and one dose of epinephrine, patient will wake up and be neurologically intact. Learner will call cardiology for cath lab activation.*

**Anticipated Management Mistakes**

1. *Failure to order aspirin: Learners often forgot to order aspirin, but as the case developed into a Vfib arrest, we had the cardiologist at the end prompt learners on the correct medications.*
2. *Not placing patient on cardiac monitors: learners sometimes did not place the patient on monitors until the patient went unresponsive. The nurse sometimes had to prompt the learners to place patient on monitor.*
3. *Reluctance to run a team: Learners sometimes forgot to assign roles. As a result, members of the team were unable to help as they were not assigned a role. During a debrief, we specifically concentrated on effective leadership in any resuscitations.*

Multimedia:


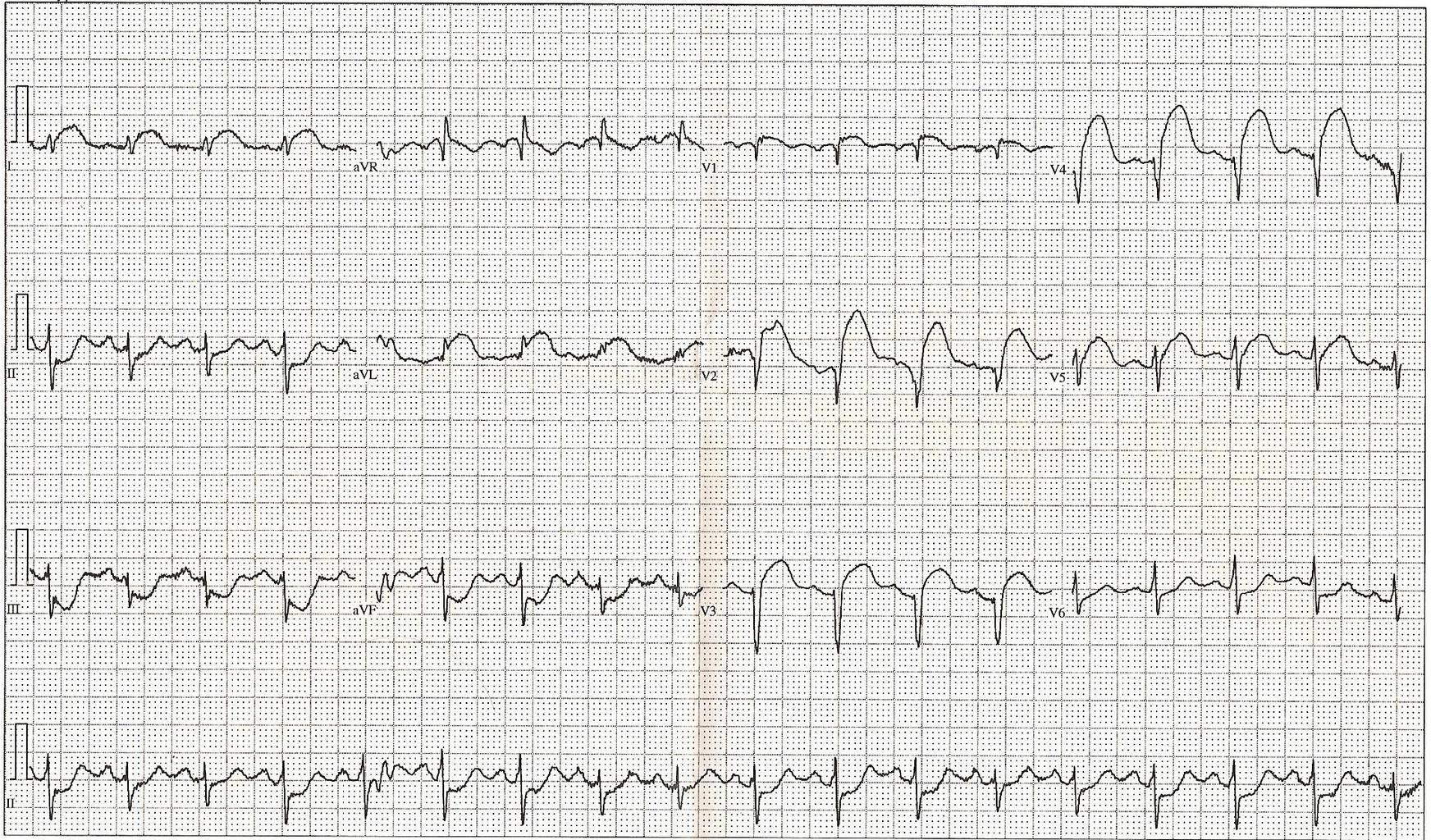


Image by [Wikimedia], retrieved from: [https://free-images.com/display/12_lead_ekg_st.html] on [12/05/2018]. Image is in the public domain.


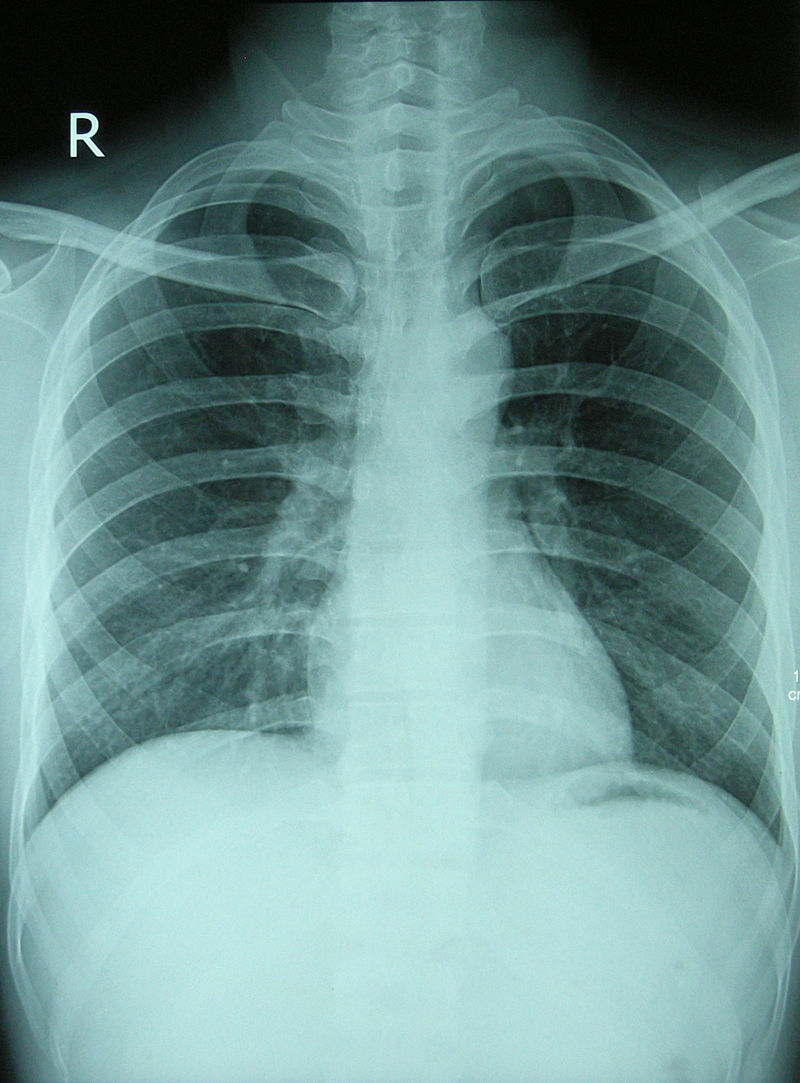


Image by [Wikimedia], retrieved from: [https://commons.wikimedia.org/wiki/File:Chest_X-ray_2346.jpg] on [10/16/19]. Image is in the public domain.

https://commons.wikimedia.org/wiki/File:Chest_X-ray_2346.jpg
