# Supplementary material for: Establishing a Low-Resource Simulation Emergency Medicine Curriculum in Nepal
Source: MedEdPORTAL. 2020 Jul 15;16:10924. doi: 10.15766/mep_2374-8265.10924 (PMC7373349; doi:10.15766/mep_2374-8265.10924)
Supplement: Supplementary file 1 — Trauma With Tension Pneumothorax.docxMyocardial Infarction With V-fib.docxPneumonia With Septic Shock.docxOrganophosphate Poisoning.docxACLS Cardiac Arrest.docxAnaphylaxis.docxTrauma With Subdural Hematoma.docxProcedure-Specific Lab.docxSimulation Curriculum Survey.docx [file mep_2374-8265.10924-s001.zip › C. Pneumonia with Septic Shock.docx]

| **Appendix C: Pneumonia with septic shock**  **SIMULATION CASE TITLE: Pneumonia with septic shock**  **AUTHORS: Alfred Wang MD** | |
| --- | --- |
| **PATIENT NAME: Nanu**  **PATIENT AGE: 61 years old**  **CHIEF COMPLAINT: Fever, cough** | |
|  | |
| **Brief narrative description of case** | *61 year old female coming in with fevers and cough for 3 days.*  *Learners are expected to recognize septic shock secondary to pneumonia and resuscitate patient appropriately and disposition the patient to the medical intensive care unit (ICU).* |
| **Primary Learning Objectives** | 1. *Demonstrate the ability to organize and lead the care team.* 2. *Formulate a differential diagnosis for shock and propose treatment options for septic shock* 3. *Demonstrate ability to escalate care after recognizing septic shock that is not responsive to fluids* 4. *Explain care to patient’s family* |
| **Critical Actions** | 1. *The learner will take lead and assign clear roles*  - *Ask for help* - *Ask for intravenous (IV) access, to have patient placed on monitor, call for nasal cannula* - *Ask for vital signs*  1. *Entertain differential diagnoses for chest pain*    1. *These may include acute coronary syndrome, pulmonary embolism, aortic dissection, cardiac tamponade* 2. *Obtain full history including past medical history, allergies and social history* 3. *Recognize sepsis as patient is febrile, hypotensive, tachycardic and tachypneic* 4. *Intervene appropriately to sepsis*  - *Address low blood pressure with fluids* - *Give patient antipyretic* - *Obtain labs: CBC, BMP, blood cultures, lactate. Consider obtaining a urine analysis and urine culture.* - *Obtain CXR*  1. *Accurately read the CXR as positive for a right lower lobe pneumonia and intervene appropriately*  - *Order appropriate antibiotics after considering patient’s allergies (potential antibiotics include fluoroquinolones, macrolides, tetracyclines)*  1. *The learner will communicate effectively with other members of the hospital system and disposition the patient appropriately*  - *Call intensive care unit and describe case succinctly*  1. *Provide effective team leadership*  - *Verbally assign roles* - *Provide specific instructions* - *Remain calm* |
| **Learner Preparation** | *No advance information needed* |

| Initial Presentation | | | |
| --- | --- | --- | --- |
| **Initial vital signs** | HR 120, BP 80/50, RR 24, Temp 39.2, O2 sat on room air 90% | | |
| **Overall Appearance** | *Patient resting in bed and intermittently coughs.* | | |
| **Actors and roles in the room at case start** | *There is a nurse in the room.*  *There is a family member in room who will provide most of the history and also show concern for patient.* | | |
| **HPI** | *Family member and patient will tell learner that the chief complain is fevers and cough for 3 days.*  *All history can be obtained from family member if asked.*  *Patient has been having a cough and fever for 3 days. She is getting weaker and more confused. She is alert and oriented x3 but sluggish to respond to questions. When asked of pain, patient will answer and they she has R sided chest pain when breathing. Family will say she has had a productive cough. Says she has not taken any medications. Patient will deny stiff neck or headache. Review of systems will be otherwise negative if asked.* | | |
| **Past Medical/Surgical History** | **Medications** | **Allergies** | **Family History** |
| Hypertension | Lisinopril | Penicillin | Non-contributory |
| **Physical Examination** | | | |
| **General** | Patient resting in bed. Looks unwell. | | |
| **HEENT** | Atraumatic/normocephalic, extraocular movement intact, conjunctiva normal, dry oral mucosa | | |
| **Neck** | Supple | | |
| **Lungs** | Decreased breath sounds in R lower lobe | | |
| **Cardiovascular** | Regular rhythm. Tachycardic. No murmurs. | | |
| **Abdomen** | Soft, Nontender. | | |
| **Neurological** | Slow to respond to questions but follows commands and answers some questions. | | |
| **Skin** | No rashes. Dry. | | |
| **GU** | Normal. | | |
| **Psychiatric** | Normal affect. | | |

| Instructor Notes - Changes and CASE Branch Points | | |
| --- | --- | --- |
| **Intervention / Time point** | **Change in Case** | **Additional Information** |
| *Learner requests CXR* | *Learner shown CXR in multimedia after a delay of 3-5 minutes.* | *CXR is available in Patan hospital but patient will have to be wheeled to get CXR.* |
| *Learner requests EKG* | *Learner shown EKG in multimedia* |  |
| *2 minutes into the case* | *BP begins decreasing if no IV access started* | *RN will alert provider: “Doctor the blood pressure is low”* |
| *5 minutes into the case* | *BP begins decreasing if no IV fluids and patient gets less responsive.* | *RN will ask learner “what should we do with the blood pressure?”* |
| *Oxygen given* | *O2 saturation increases to 99%* |  |
| *2L of fluid given.* | *BP remains around 90/40.* | *Family will ask learner for an update.* |
| *If no vasopressors started after fluids.* | *BP continues to decrease: will drop to 60 systolic.* | RN will ask learner to address the low blood pressure. |
| *Patient given penicillin* | *Patient becomes unresponsive. BP will be unable to obtain. Person running the simulation lab will inform learner patient is stridorous and face is swelling.* | *Case will end.* |
| *Correct antibiotics given and vasopressors started* | *BP improves to 120/60, HR 80.* |  |

**Ideal Scenario Flow**

*The learners enter the room to find a 61 year old woman who looks unwell with her family member. They immediately ask the nurse to start an IV, place the patient on the monitor and obtain vitals. They will recognize that the patient is hypoxic and hypotensive. Supplemental oxygen is provided, and IV fluid bolus is ordered. After learner completes history and physical, learner recognizes that the blood pressure has not improved with fluids. Learner orders appropriate antibiotics. Chest X ray will demonstrate a R lower lobe pneumonia. Laboratory studies will demonstrate an elevated WBC and lactate (otherwise normal). Learner orders vasopressors to treat septic shock. The vitals will improve. Learner also updates family with the plan and dispositions patient to the medical ICU.*

**Anticipated Management Mistakes**

1. *Not obtaining vitals soon enough: We found that some learners had a delay in diagnosis in septic shock due to not obtaining the blood pressure early enough. We had the nurse prompt the learners if the blood pressure was not obtained.*
2. *Failure to start vasopressors when fluids not improving blood pressure: Most of the time, this was due to learners not obtaining repeat vitals so they did not recognize that the blood pressure had not improved. The family member would ask for an update and ask why the patient was not improving with treatment to prompt learners to get repeat vitals.*
3. *Not obtaining a complete history: During our cases no patients died due to anaphylaxis, but we foresee that some learners may forget to get a complete history and anchor too early. This will also manifest in a lack of differential diagnoses as well. Debriefing involved reviewing differential diagnoses for chest pain.*

Multimedia


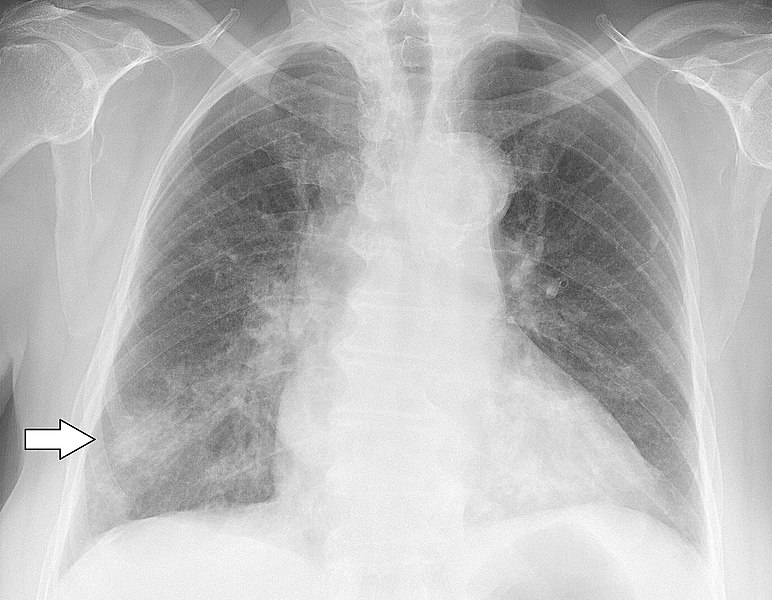


“Image by [Mikael Haggstrom], retrieved from: [https://commons.wikimedia.org/wiki/File:Chest_X-ray_in_influenza_and_Haemophilus_influenzae_-_annotated.jpg] on [12/05/18]. Creative Commons License associated: [https://creativecommons.org/publicdomain/zero/1.0/deed.en].”


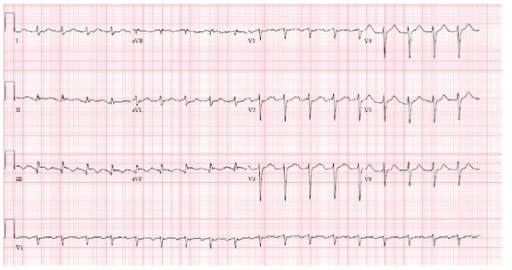


Image by [Department of Internal Medicine, University of Floria College of Medicine], retrieved from: [https://openi.nlm.nih.gov/detailedresult.php?img=PMC4055418_CRIC2014-120607.001&req=4] on [12/07/18]. Creative Commons License associated: [https://creativecommons.org/licenses/by/3.0/]
