# Supplementary material for: Establishing a Low-Resource Simulation Emergency Medicine Curriculum in Nepal
Source: MedEdPORTAL. 2020 Jul 15;16:10924. doi: 10.15766/mep_2374-8265.10924 (PMC7373349; doi:10.15766/mep_2374-8265.10924)
Supplement: Supplementary file 1 — Trauma With Tension Pneumothorax.docxMyocardial Infarction With V-fib.docxPneumonia With Septic Shock.docxOrganophosphate Poisoning.docxACLS Cardiac Arrest.docxAnaphylaxis.docxTrauma With Subdural Hematoma.docxProcedure-Specific Lab.docxSimulation Curriculum Survey.docx [file mep_2374-8265.10924-s001.zip › D. Organophosphate Poisoning.docx]

| **Appendix D: Organophosphate poisoning**  **SIMULATION CASE TITLE: Organophosphate poisoning**  **AUTHORS: Alfred Wang MD** | |
| --- | --- |
| **PATIENT NAME: Manish**  **PATIENT AGE: 30 years old**  **CHIEF COMPLAINT: vomiting** | |
|  | |
| **Brief narrative description of case** | *30 year old farmer comes in with vomiting, salivation and respiratory distress that started earlier today after fertilizing his crops.*  *Learners are expected to recognize organophosphate poisoning and recognize the importance of personal protective equipment (PPE), decontamination, and resuscitate the patient.* |
| **Primary Learning Objectives** | 1. *Demonstrate the ability to organize and lead the care team.* 2. *Recognize organophosphate poisoning and illustrate ability to prioritize scene safety with immediate decontamination* 3. *Define treatment options used for organophosphate poisoning* 4. *Demonstrate contact of appropriate consultants for disposition* |
| **Critical Actions** | 1. *The learner will take lead and assign clear roles*  - *Ask for help* - *Ask for intravenous (IV) access, to have patient placed on monitor, call for nasal cannula* - *Ask for vital signs*  1. *Promptly perform focused History and Physical Exam*  - *Discover complaints of nausea, abdominal cramping, tearing of his eyes, excessive salivation, sweating profusely and shortness of breath in a farmer*  1. *Recognize potential organophosphate poisoning and immediately ensure team safety*  - *Decontaminate patient by removing clothes and washing patient to remove remaining toxin* - *Give reusable fabric gowns, gloves, masks to all healthcare workers involved in patient’s care*  1. *If learner orders EKG, recognize cause of sinus bradycardia as cholinergic poisoning* 2. *Order atropine and pralidoxime as definitive treatment of organophosphate poisoning.*  - *Doses utilized at Patan hospital are 1-3 mg atropine IV push doses and 1g of pralidoxime over 1 hour* - *Continue push doses of atropine until patient clinically improves, which means improvement in vitals as well as decrease in hypersalivation* - *Recognize that to obtain proper treatment, high doses of atropine will be needed* - *Recognize patient will need an atropine drip ordered once stabilized with bolus dosing*  1. *The learner will communicate effectively with other members of the hospital system and disposition the patient appropriately*  - *Call intensive care unit and describe case succinctly*  1. *Provide effective team leadership*  - *Verbally assign roles* - *Provide specific instructions* - *Remain calm* |
| **Learner preparation** | *There is no pre-reading required.*  *Before case starts, learner will be told that the patient is a 30 year old farmer coming in with vomiting after fertilizing his crops earlier this morning.* |

| Initial Presentation | | | |
| --- | --- | --- | --- |
| **Initial vital signs** | HR 50, BP 100/60, RR 25, Temp 36.6, Pulse ox on room air 90% | | |
| **Overall Appearance** | *Patient salivating and in mild respiratory distress* | | |
| **Actors and roles in the room at case start** | *A nurse in room to provide help.* | | |
| **HPI** | *Patient will tell learner that he is here due to vomiting.*  *All information can be given by patient when learner asks for it.*  *Patient says he started vomiting earlier this morning. He also says he has been salivating more and has had difficult breathing. He says when he woke up today he went to fertilize his crops but forgot to bring gloves. He says yesterday he was feeling his normal health. He endorses nausea, abdominal cramping, tearing of his eyes, excessive salivation, sweating profusely and shortness of breath. Review of systems is otherwise negative.* | | |
| **Past Medical/Surgical History** | **Medications** | **Allergies** | **Family History** |
| None | None | None | Non-contributory |
| **Physical Examination** | | | |
| **General** | Patient salivating and holding a bag and vomiting intermittently. | | |
| **HEENT** | Constricted pupils. Reactive to light bilaterally. Excessive tearing in bilateral eyes. Conjunctiva normal. Extraocular movements intact. | | |
| **Neck** | Supple. | | |
| **Lungs** | Bilateral wheezing. Patient tachypneic. | | |
| **Cardiovascular** | Bradycardic. Regular rhythm. | | |
| **Abdomen** | Soft, mild diffuse tenderness to palpation, hyperactive bowel sounds | | |
| **Neurological** | No focal neurologic deficits. | | |
| **Skin** | Sweaty. | | |
| **GU** | Normal | | |
| **Psychiatric** | Normal affect | | |

| Instructor Notes - Changes and CASE Branch Points | | |
| --- | --- | --- |
| **Intervention / Time point** | **Change in Case** | **Additional Information** |
| *Learner requests EKG* | *EKG in multimedia shown to learner* |  |
| *Learner requests CXR* | *CXR in multimedia shown to learner after a delay of 3-5 minutes.* | *CXR is available in Patan hospital but patient will have to be wheeled to get CXR.* |
| *2 minutes into case* | *If learner has not asked for decontamination and for personal protective equipment for the team, the nurse in room will start saying she feels sick and wants to vomit* | *RN becomes sick.* |
| *Learner asks for patient care by nurse (IV access, monitor leads placed)* |  | *Nurse asks provider if she should be touching the patient since she is getting sicker and starts to actively vomit.* |
| *With atropine and pralidoxime administered* | *Patient says he feels a little better but still sweating and hyper-salivating. If not administered, patient will say that he is feeling worse and HR will drop to 30s.* |  |
| *With 10 mg IV atropine administered* | *Patient vitals improve. BP 100/72, RR 18, HR 100, Tempt 37.8, Oxygen sat on room air 100%* |  |
| *10 minutes into case* | *If nurse was exposed, he/she will be unavailable to help in the rest of the case. The person running the simulation will end the case* | *The person running the simulation will inform the learner that he/she is also getting symptoms.* |

**Ideal Scenario Flow**

*The learners enter the room to find a patient sweating, hyper-salivating and tachypneic. Learner recognizes early that this is organophosphate poisoning and asks for all team members to wear personal protective equipment, which in Patan hospital is reusable fabric gowns and gloves and paper masks. The learner asks for patient to be decontaminated prior to full evaluation. After decontamination, learner completes a full history and physical exam. Work-up is obtained which is optional. If a chest x-ray is obtained, chest x-ray should demonstrate diffuse pulmonary edema. If EKG obtained, sinus bradycardia is demonstrated. Learner asks for appropriate treatment medications: atropine and pralidoxime. Once patient starts to improve, learner will arrange for patient admission to medical ICU.*

**Anticipated Management Mistakes**

1. *Delay in recognition of diagnosis: We found that learners recognized the diagnosis immediately but forgot scene safety. In this case, the nurse became sick which prompted learners to decontaminate the patient and obtain protective equipment for the rest of the team.*
2. *Failure to recognize the treatment for organophosphate poisoning: All learners gave our simulated patient atropine but some forgot to give pralidoxime. This required prompting by having the patient decompensate further. All learners did continue giving atropine until patient clinically improved.*
3. *Uncertainty regarding final disposition: Although ICU was readily available in Patan hospital, some learners wanted to admit to the medical floor. The simulation leader who acted as the accepting provider would prompt learners that the patient likely needed airway management and an atropine drip and required ICU care.*

Multimedia


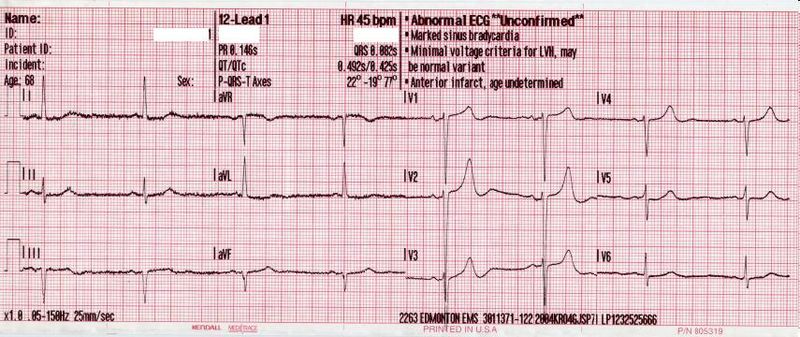


“Image by [Glenlarson], retrieved from: [https://commons.wikimedia.org/wiki/File:12_lead_sinus_bradycardia.JPG] on [12/05/18]. Image is in the public domain.”


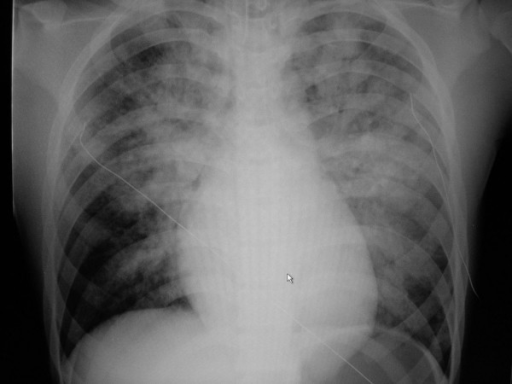

“Image by [Department of Pediatrics, Children Medical Center, Chang Gung Memorial Hospital, Chang Gung University College of Medicine], retrieved from: [https://openi.nlm.nih.gov/detailedresult.php?img=PMC2637259_1757-1626-2-52-1&req=4] on [12/07/18]. Creative Commons License associated: [https://creativecommons.org/licenses/by/2.0/].”
