# Supplementary material for: Establishing a Low-Resource Simulation Emergency Medicine Curriculum in Nepal
Source: MedEdPORTAL. 2020 Jul 15;16:10924. doi: 10.15766/mep_2374-8265.10924 (PMC7373349; doi:10.15766/mep_2374-8265.10924)
Supplement: Supplementary file 1 — Trauma With Tension Pneumothorax.docxMyocardial Infarction With V-fib.docxPneumonia With Septic Shock.docxOrganophosphate Poisoning.docxACLS Cardiac Arrest.docxAnaphylaxis.docxTrauma With Subdural Hematoma.docxProcedure-Specific Lab.docxSimulation Curriculum Survey.docx [file mep_2374-8265.10924-s001.zip › E. ACLS Cardiac Arrest.docx]

| **Appendix E: Cardiac arrest**  **SIMULATION CASE TITLE: ACLS Cardiac arrest**  **AUTHORS: Alfred Wang MD** | |
| --- | --- |
| **PATIENT NAME: Krishna**  **PATIENT AGE: 54 years old**  **CHIEF COMPLAINT: chest pain** | |
|  | |
| **Brief narrative description of case** | *54 year old presenting to emergency department from home with chest pain for 3 hours.*  *Learners are expected to recognize pulseless electrical activity (PEA), ventricular fibrillation (Vfib), and stable supraventricular tachycardia (SVT) and provide appropriate management.* |
| **Primary Learning Objectives** | 1. *Demonstrate the ability to organize and lead the care team* 2. *Distinguish the different arrhythmias on the cardiac monitor* 3. *Apply ACLS skills in the resuscitation* 4. *Discuss post-cardiac arrest care, including laboratory and EKG interpretation* |
| **Critical Actions** | 1. *The learner will take lead and assign clear roles*  - *Ask for help* - *Ask for intravenous (IV) access, to have patient placed on monitor, call for nasal cannula* - *Ask for vital signs*  1. *Recognize pulseless electrical activity (PEA)* 2. *Manage PEA arrest per Advance Cardiovascular Life Support (ACLS) protocols*  - *Perform CPR in 2-minute cycles with pulse/rhythm checks in between* - *Give intravenous epinephrine (1mg 1:10,000) every 3-5 minutes.* - *Discuss reversible causes of PEA arrest:* - *These can include Hypoxia, Acidosis, Hyperkalemia, Hypokalemia, Hypothermia, Tamponade, Tension pneumothorax, Coronary or pulmonary thrombosis, Toxins*  1. *Recognize Vfib arrest.* 2. *Manage Vifb arrest per ACLS protocols*  - *Ask for defibrillator and defibrillate patient* - *Continue CPR with pulse and rhythm checks every 2 minutes* - *Give 1mg of (1:10,000) epinephrine every 3-5 minutes*  1. *Recognize stable SVT* 2. *Manage stable SVT per ACLS protocols*  - *Consider vagal maneuvers (these may include carotid sinus massage, Valsalva maneuver)* - *Consider adenosine 6 mg intravenous. May give second dose at 12 mg.*  1. *Discuss possible post-cardiac arrest care:*  - *This may include 12-lead EKG; lab work such as troponin, BMP, CBC; consider definitive airway such as intubation if patient was not neuro-intact* |
| **Learner Preparation** | *No advance information required.* |

| Initial Presentation | | | |
| --- | --- | --- | --- |
| **Initial vital signs** | T 37, HR 80, BP 140/80, RR 12, Oxygen saturation on room air 99% | | |
| **Overall Appearance** | *Ill appearing diaphoretic obese male clutching his chest* | | |
| **Actors and roles in the room at case start** | *There is a nurse to help assist.* | | |
| **HPI** | *54 year old male coming in with chest pain that occurred 3 hours prior to arrival when he is working in his shop. He says he feels nauseous and describes his chest pain as crushing without radiation. Before he is able to give any further information, patient becomes unresponsive.* | | |
| **Past Medical/Surgical History** | **Medications** | **Allergies** | **Family History** |
| Unable to obtain | Unable to obtain | Unable to obtain | Unable to obtain |
| **Physical Examination** | | | |
| **General** | Diaphoretic obese male | | |
| **HEENT** | Pupils equal and reactive. | | |
| **Neck** | Supple | | |
| **Lungs** | Clear bilaterally | | |
| **Cardiovascular** | If pre-arrest, regular rate and rhythm. If during arrest, depend on scenario. | | |
| **Abdomen** | Soft. No bruising. | | |
| **Neurological** | If pre-arrest, alert and oriented and follows commands briskly. | | |
| **Skin** | Normal | | |
| **GU** | Normal | | |
| **Psychiatric** | Normal affect | | |

| Instructor Notes - Changes and CASE Branch Points | | |
| --- | --- | --- |
| **Intervention / Time point** | **Change in Case** | **Additional Information** |
| *30 seconds into case* | *Patient becomes unresponsive.* | *RN alerts provider that patient is unconscious* |
| *30 seconds into case* | Monitor demonstrates no electrical activity. If pulse is checked by learner, no pulse is felt. |  |
| If roles are not assigned during code. |  | RN asks provider if he/she wants to assign roles |
| After 2 cycles of CPR and 1 dose of epinephrine given | *Monitor changes into Vfib.* |  |
| *If patient does not receive shocks* | *Patient continues to be in Vfib.* | *RN asks provider if he/she wants to defibrillate.* |
| *2 cycles of CPR, 1 shock, 1 dose of epinephrine given.* | *Monitor changes into SVT. If pulse is checked by learner, pulse is felt.* |  |
| *If learner does not check pulse and shocks patient instead.* | *Patient screams out in pain.* |  |
| *If adenosine given.* | *Patient reverts to normal sinus rhythm. Vitals: BP 140/80, RR 14, HR 100, room air saturation of 100%.* |  |

**Ideal Scenario Flow**

*The learners enter the room to find a patient who is ill appearing, clutching his chest. They immediately ask the nurse to place the patient on a monitor, obtain IV access and obtain vitals. The patient tells a brief history and then becomes unconscious. The learner then assigns team roles (if only one nurse, then nurse will perform all roles/ but if there are more than one people- one person can record, one can do CPR, one can manage the airway, one can give mediations). The learner recognizes that that the initial rhythm is PEA arrest and asks for high-quality CPR to be performed. Every two minutes he asks for a pulse check and gives epinephrine every 3-5 minutes. Learner also verbalizes potential reversible causes of PEA. After two cycles of CPR and 1 dose of epinephrine, the learner recognizes that the rhythm now has changes into Vfib. The learner asks to resume CPR and does pulse checks every 2 minutes. During these pulse checks, the rhythm remains Vfib and the learner clears before defibrillating the patient. Every 3-5 minutes, learner gives epinephrine. After 2 cycles of CPR, 1 shock and 1 dose of epinephrine, the rhythm becomes SVT and patient has a pulse. Vital signs are normal as well. The learner should recognize SVT and manage it with vagal maneuvers and adenosine. The patient at this time will revert to normal sinus rhythm and be neuro intact. The learner will call cardiology for ultimate disposition and ask for post-cardiac arrest care such as a 12-lead EKG and labs.*

**Anticipated Management Mistakes**

1. *Delay in placing patient on monitor: We found that because how rapidly the patient decompensates, many learners had not placed the patient on the monitor. We were able to emphasize during our debriefing that it was important to get a patient on a monitor as soon as possible.*
2. *Delay in assigning roles and leading the room: We found that learners often did not assign roles and the nurse had to prompt learners. In our scenario, the team leader did have a team of other learners to help and often assigning roles was delayed. During our debriefing, we discussed the importance of communication in a cardiac arrest resuscitation.*
3. *Failure to recognize the steps post-cardiac arrest: Most learners recognized the next steps after cardiac arrest but some needed prompting regarding labs and ultimate disposition. We found it helpful to have the nurse prompt learners for further care.*
