# Supplementary material for: Establishing a Low-Resource Simulation Emergency Medicine Curriculum in Nepal
Source: MedEdPORTAL. 2020 Jul 15;16:10924. doi: 10.15766/mep_2374-8265.10924 (PMC7373349; doi:10.15766/mep_2374-8265.10924)
Supplement: Supplementary file 1 — Trauma With Tension Pneumothorax.docxMyocardial Infarction With V-fib.docxPneumonia With Septic Shock.docxOrganophosphate Poisoning.docxACLS Cardiac Arrest.docxAnaphylaxis.docxTrauma With Subdural Hematoma.docxProcedure-Specific Lab.docxSimulation Curriculum Survey.docx [file mep_2374-8265.10924-s001.zip › F. Anaphylaxis.docx]

| **Appendix F: Anaphylaxis**  **SIMULATION CASE TITLE: Anaphylaxis**  **AUTHORS: Alfred Wang MD** | |
| --- | --- |
| **PATIENT NAME: Solomon**  **PATIENT AGE: 46 years old**  **CHIEF COMPLAINT: Rash, shortness of breath** | |
|  | |
| **Brief narrative description of case** | *46 year old male coming in with itchy rash and shortness of breath after taking his first dose of azithromycin an hour prior to arrival.*  *Learners are expected to recognize anaphylaxis, give appropriate medications, and intubate patient.* |
| **Primary Learning Objectives** | 1. *Demonstrate the ability to organize and lead the care team.* 2. *Demonstrate ability to diagnose anaphylaxis and apply this knowledge to the correct management of anaphylaxis* 3. *Correctly implement rapid sequence intubation techniques to perform emergency intubation* 4. *Assess patient’s clinical status as critical and admit to the intensive care unit* |
| **Critical Actions** | 1. *Promptly recognize critical illness*    1. *Recruit multiple healthcare workers to assist*    2. *Obtain intravenous (IV) access, place on monitor, apply oxygen, and obtain vital signs* 2. *Promptly perform focused History and Physical Exam*    1. *Discover complaints of dyspnea, itching rash, and facial swelling*    2. *Discover association with medication use*    3. *Identify tachycardia, wheezing, and urticaria on exam* 3. *Recognize anaphylaxis and treat with appropriate dose/route of epinephrine*    1. *Verbalize diagnosis*    2. *Administer 300-500mcg of 1:1000 epinephrine IM* 4. *Recognize need for intubation*    1. *Verbalize impending airway failure*    2. *Verbalize impending breathing failure despite maximal non-invasive methods* 5. *Recognize need for backups for possible difficult intubation*    1. *Verbalize anticipated difficult airway*    2. *Collect adjuncts and difficult airway equipment (eg NPA, OPA, stylet, bougie, scalpel/cric kit)* 6. *Promptly lead team in successful intubation*    1. *Attempt to pre-oxygenate with non-rebreather at high flow rate*    2. *Assemble equipment, at minimum:*        1. *Bag-valve-mask (BVM) attached to O2, suction, laryngoscope (confirmed functional), endotracheal tube (size 7.0-8.0, confirmed intact balloon), stethoscope*    3. *Recognize and utilize an acceptable sedative at correct dose for RSI:*       1. *E.g. Etomidate, 0.2-0.4mg/kg IV, Ketamine, 1-2mg/kg IV, or Propofol 1-3mg/kg IV*    4. *Perform endotracheal intubation, and verbalize confirmation with multiple methods (e.g. direct visualization, fog in tube, absent epigastric sounds, bilateral chest rise, bilateral breath sounds) that must include end-tidal CO2 colorimetry or capnography*    5. *Ensure tube not displaced*       1. *Do not release ETT until securely fixed with tape or securement device*       2. *Ensure patient secured with non-violent restraints after procedure* 7. *Update family once initial stabilization complete*    1. *Discuss presentation, evaluation, current diagnosis, severity and next steps* 8. *Promptly admit to medical intensive care unit (ICU) after patient is stabilized* 9. *Provide effective team leadership*    1. *Verbally assign roles*    2. *Provide specific instructions*    3. *Remain calm* |
| **Learner Preparation** | *No advance information required.* |

| Initial Presentation | | | |
| --- | --- | --- | --- |
| **Initial vital signs** | HR 105, BP 90/50, RR 24, Oxygen saturation on room air 91%, T 36.9 | | |
| **Overall Appearance** | *A 46 year old male who has swelling around face and slightly tachypneic* | | |
| **Actors and roles in the room at case start** | *There is a nurse in the room.* | | |
| **HPI** | *Patient will tell provider that he is here for shortness of breath, an itchy rash, and swelling around the face.*  *When asked, patient will tell provider that this happened an hour after taking his first dose of azithromycin. The symptoms have been gradually worsening for about 2-3 hours. He was recently diagnosed with pneumonia. He denies any fevers or pain anywhere. He has never felt like this before. Denies any diarrhea, nausea or vomiting. Review of systems is otherwise negative.* | | |
| **Past Medical/Surgical History** | **Medications** | **Allergies** | **Family History** |
| Chronic obstructive pulmonary disease | Albuterol | None | Non-contributory |
| **Physical Examination** | | | |
| **General** | Patient slightly tachypneic but comfortable. Speaking full sentences. Has swelling around lips and face. | | |
| **HEENT** | Pupils equal and reactive. Full extraocular movements. No swelling in mouth. | | |
| **Neck** | Supple. | | |
| **Lungs** | Bilateral wheezing. | | |
| **Cardiovascular** | Tachycardic. Regular rhythm. No murmurs. Brisk and equal distal pulses. | | |
| **Abdomen** | Soft, non-tender, non-distended | | |
| **Neurological** | Alert and oriented. Able to answer all questions. Non-focal neurological exam. | | |
| **Skin** | Diffuse urticaria. | | |
| **GU** | Normal | | |
| **Psychiatric** | Normal affect | | |

| Instructor Notes - Changes and CASE Branch Points | | |
| --- | --- | --- |
| **Intervention / Time point** | **Change in Case** | **Additional Information** |
| *5 minutes into the case and epinephrine has not been given* | *BP begins decreasing and patient becomes more tachypneic* | *Patient has active stridor and asks provider “why can’t I breathe?” RN asks provider “what medication can help?”* |
| *Provider asks for epinephrine* |  | *RN asks for dose and route.* |
| *2 minutes after epinephrine is given* | *Patient becomes more tachypneic and oxygen saturation drops to 75%.* | *RN tells provider that oxygen saturation is dropping.* |
| *More epinephrine given but has not been intubated* | *Patient HR will continue to increase and BP will increase but oxygen saturation will continue to drop until intubation.* |  |
| *Provider decides to intubate* |  | *RN will ask for direction- for example medications. Will ask provider if he wants any backup items if provider does not verbalize backups.* |
| *After intubation* | *O2 saturation will increase to 100%. BP improve to 105/70. HR to 110.* |  |

**Ideal Scenario Flow**

*The learners enter the room to find the patient who is slightly tachypneic with diffuse urticaria and swelling around the lips. They immediately ask for IV access, to have the patient placed on monitors, and ask for a first set of vital signs. Once they notice O2 saturation is low, they should ask for oxygen. After a brief history and physical, provider realizes patient is anaphylactic and ask nurse for IM epinephrine 300-500 micrograms. The provider can ask for other medications such as steroids, albuterol, ranitidine. The patient’s respiratory distress does not improve after medications and oxygen saturation continues to drop. The learner recognizes the need for intubation and asks the team to prepare for intubation. This will entail asking for suction, supplies, medications. If the learner does not ask for backups, the RN will ask for possible back up options if intubation fails. The patient is successfully intubated and the saturations improve. The learner dispositions the patient to the medical ICU.*

**Anticipated Management Mistakes**

1. *Not intubating soon enough: We found that because our mannikin did not allow for intubation, we had to prompt our learners to intubate the airway trainer which was set up by the head of the bed. This is more likely due to the lack of resources but with prompting, learners intubated successfully and continued the case.*
2. *Not knowing the correct dosage for epinephrine: Because in the actual emergency department, most nurses know the dosages, the learners sometimes did not know the dosage of epinephrine when asked. We made this a teaching point during debriefing.*
3. *Failure to recognize the need for backups for intubation: None of the learners had done a cricothyrotomy and did not know what instruments were needed for the backup. We allowed learners to just ask for a cricothyrotomy kit nearby but did not ask for the specifics. Many learners did request further procedural skills such as cricothyrotomy, so we plan to create further simulations that addresses these skills.*
