# Supplementary material for: Establishing a Low-Resource Simulation Emergency Medicine Curriculum in Nepal
Source: MedEdPORTAL. 2020 Jul 15;16:10924. doi: 10.15766/mep_2374-8265.10924 (PMC7373349; doi:10.15766/mep_2374-8265.10924)
Supplement: Supplementary file 1 — Trauma With Tension Pneumothorax.docxMyocardial Infarction With V-fib.docxPneumonia With Septic Shock.docxOrganophosphate Poisoning.docxACLS Cardiac Arrest.docxAnaphylaxis.docxTrauma With Subdural Hematoma.docxProcedure-Specific Lab.docxSimulation Curriculum Survey.docx [file mep_2374-8265.10924-s001.zip › G. Trauma with Subdural Hematoma.docx]

| **Appendix G: Trauma with large subdural hematoma (SDH)**  **SIMULATION CASE TITLE: Trauma with SDH**  **AUTHORS: Alfred Wang MD** | |
| --- | --- |
| **PATIENT NAME: Rami**  **PATIENT AGE: 26 years old**  **CHIEF COMPLAINT: Pedestrian hit by car** | |
|  | |
| **Brief narrative description of case** | *26 year old brought in by family after being hit by a car going unknown speed. Patient is unresponsive on arrival with clear head trauma.*  *Learners are expected to perform a primary survey- recognizing patient needs to be intubated, and recognize signs of intracranial hemorrhage and perform maneuvers to decrease intracranial pressure.* |
| **Primary Learning Objectives** | 1. *Demonstrate the ability to organize and lead the care team.* 2. *Demonstrate the ability to perform a primary survey and refine differential diagnosis of a patient with unresponsiveness after head trauma* 3. *Recognize need for intubation and organize the team to assist the intubation* 4. *Design an appropriate treatment plan after recognition of increased intracranial pressure* |
| **Critical Actions** | 1. *Promptly recognize critical illness*    1. *Recruit multiple healthcare workers to assist*    2. *Obtain intravenous (IV) access, place on monitor, apply oxygen, and obtain vital signs*    3. *Order stat fingerstick glucose in patient with altered mental status* 2. *Promptly perform primary trauma assessment, and intervene*    1. *Assess airway, breathing, circulation while protecting c-spine (via assistant)*    2. *Briefly assess neurologic disability via Glasgow coma score or AVPU score* 3. *Recognize need for intubation*    1. *Verbalize that patient is not protecting their airway* 4. *Promptly lead team in successful intubation*    1. *Attempt to pre-oxygenate with non-rebreather at high flow rate*    2. *Assemble equipment, at minimum:*        1. *Bag-valve-mask (BVM) attached to O2, suction, laryngoscope (confirmed functional), endotracheal tube (size 7.0-8.0, confirmed intact balloon), stethoscope, ETCo2 device*    3. *Ensure cervical spine precautions throughout procedure (via assistant)*    4. *Recognize and utilize an acceptable sedative at correct dose for RSI:*       1. *E.g. Etomidate, 0.2-0.4mg/kg IV, or Propofol 1-3mg/kg IV*    5. *Perform endotracheal intubation, and verbalize confirmation with multiple methods (e.g. direct visualization, fog in tube, absent epigastric sounds, bilateral chest rise, bilateral breath sounds)*    6. *Ensure tube not displaced*       1. *Do not release ETT until securely fixed with tape or securement device* 5. *Perform appropriate post-intubation care*    1. *Provide appropriate post-intubation sedation*       1. *E.g. benzodiazepine or propofol drips, plus fentanyl drip*    2. *Obtain and interpret STAT post-procedural CXR to confirm tube position* 6. *Promptly perform focused History and Physical Exam*    1. *Discover patient was hit by a car if not already noted*    2. *Discover scalp hematoma if not already noted*    3. *Discover pupillary asymmetry if not already noted on primary survey*    4. *Complete secondary exam, to include whole-body exposure and exam* 7. *Promptly recognize intracranial injury with concern for impending herniation, and implement ICP lowering maneuvers*     1. *Verbalize pupil asymmetry as sign of impending herniation*    2. *Elevate head of bed while maintaining spinal precautions*    3. *Verbalize consideration of hyperosmolar therapy (e.g. hypertonic saline) and hyperventilation* 8. *Promptly obtain appropriate specialty consultation*    1. *STAT page neurosurgery and trauma for immediate consultation* 9. *Order non-contrasted head CT after initial stabilization of patient with concern for severe intracranial injury* 10. *Update family once initial stabilization complete*     1. *Discuss presentation, evaluation, current diagnosis, severity and next steps* 11. *Promptly admit to intensive care unit (ICU) after completion of specialty consultation and initial patient stabilization* 12. *Provide effective team leadership*     1. *Verbally assign roles*     2. *Provide specific instructions*     3. *Remain calm* |
| **Learner Preparation** | *No advance information is given* |

| Initial Presentation | | | |
| --- | --- | --- | --- |
| **Initial vital signs** | HR 140, BP 110/60, O2 sat on room air 88%, Temp 37, RR 25 | | |
| **Overall Appearance** | *Patient with a large hematoma around R temporal region. Not responsive.* | | |
| **Actors and roles in the room at case start** | *There is a nurse (RN) to assist. There is a worried family member in room who will provide the history.* | | |
| **HPI** | *The family member will tell provider that the patient was hit by a car when walking down a busy street 10 minutes ago. Family member was with him and patient was unresponsive after being hit. Family member was able to carry the patient and put him in a taxi and come to the hospital.*  *If asked, family member will tell this information:*  *Patient was recently healthy prior to this.* | | |
| **Past Medical/Surgical History** | **Medications** | **Allergies** | **Family History** |
| None | None | None | Non-contributory |
| **Physical Examination** | | | |
| **General** | Unresponsive. Large R temporal hematoma. | | |
| **HEENT** | Pupils: L pupils is 5 and unreactive, R is 2 and reactive. Large R temporal hematoma. No lacerations. No raccoon eyes. Tympanic membranes are normal. Oral mucosa is normal. | | |
| **Neck** | No signs of obvious trauma. | | |
| **Lungs** | Clear bilaterally | | |
| **Cardiovascular** | Sinus tachycardia. Brisk and equal distal pulses. | | |
| **Abdomen** | Soft, non-tender, non-distended. | | |
| **Neurological** | GCS of 3. | | |
| **Skin** | No bruising besides large R temporal hematoma. | | |
| **GU** | Normal. | | |
| **Psychiatric** | Unresponsive | | |

| Instructor Notes - Changes and CASE Branch Points | | |
| --- | --- | --- |
| **Intervention / Time point** | **Change in Case** | **Additional Information** |
| *Learner asks for pupil exam* | *Learner given picture of pupils in multimedia* |  |
| *Intubation not performed within 5 minutes of case* | *O2 saturation drops to 60%* | *RN alerts provider on decreasing oxygen saturations.* |
| *Intubation done without cervical-spine protection* |  | *RN asks provider: “should we do something about his neck?”* |
| *After intubation* | *O2 increases to 100%* |  |
| *2 minutes of intubation and no medications or maneuvers given* | *Both pupils become fixed and dilated* | *RN alerts provider that both pupils are now fixed and dilated* |

**Ideal Scenario Flow**

*The learners enter the room to find the patient lying on a bed with a worried family member at the bedside. The patient asks for IV access, monitor placement, oxygen, and a set of vitals signs. Learner collects a quick history and immediately moves to perform a primary survey. The airway is not intact, so the learner directs the team for an intubation with appropriate cervical-spine protection. Learner also discovers that the pupils are not equal and combined with the large R temporal hematoma, is concerned about an intra-cranial bleed. Learner asks for appropriate management to decrease ICP (which can include raising the head of the bed, hypertonic saline, hyperventilating the patient) and orders labs/imaging (which can include CBC/BMP/coagulation panel/VBG/type and screen/non-contrast CT scan, chest XR, etc). None of the labs/imaging return in time. The learner then calls neurosurgery for definitive management and asks for trauma surgery to admit to the intensive care unit. The learner updates family with the plan.*

**Anticipated Management Mistakes**

1. *Not updating the patient’s family: Learners can forget to update the worried family members in the room as they are caught up managing the sick patient. We used this case to debrief on that updating family is part of any patient’s management.*
2. *Not protecting the cervical spine: In a lower resource setting such as Patan hospital, there are no pre-hospital providers, so it is unlikely to have a patient brought in with a cervical collar placed already. As Patan hospital did not have cervical collars, the learner had to direct a team member to hold cervical spine protection during the intubation.*
3. *Not performing basic maneuvers to decrease ICP: All learners recognized the diagnosis of a possible intra-cranial bleed, but some did not remember basic maneuvers to treat this. During our debriefing we were able to go through the medical management for such a patient.*

**Multimedia**


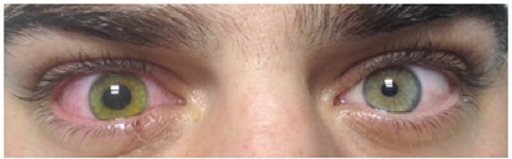


“Image by [Vardinoyiannion Eye Institute of Crete], retrieved from: [https://openi.nlm.nih.gov/detailedresult.php?img=PMC4171036_CRIOPM2014-487860.001&req=4] on [12/07/18]. Creative Commons License associated: [https://creativecommons.org/licenses/by/3.0/].”
