# Supplementary material for: Establishing a Low-Resource Simulation Emergency Medicine Curriculum in Nepal
Source: MedEdPORTAL. 2020 Jul 15;16:10924. doi: 10.15766/mep_2374-8265.10924 (PMC7373349; doi:10.15766/mep_2374-8265.10924)
Supplement: Supplementary file 1 — Trauma With Tension Pneumothorax.docxMyocardial Infarction With V-fib.docxPneumonia With Septic Shock.docxOrganophosphate Poisoning.docxACLS Cardiac Arrest.docxAnaphylaxis.docxTrauma With Subdural Hematoma.docxProcedure-Specific Lab.docxSimulation Curriculum Survey.docx [file mep_2374-8265.10924-s001.zip › H. Procedure-Specific Lab.docx]

| **Appendix H: Procedure specific lab: airway lab**  **SIMULATION CASE TITLE: Airway lab**  **AUTHORS: Alfred Wang MD** | |
| --- | --- |
| **PATIENT NAME: Ahmed**  **PATIENT AGE: 26 year old**  **CHIEF COMPLAINT: None** | |
|  | |
| **Brief narrative description of case** | *Patient presents unresponsive and needs to be intubated.*  *Learners are expected to lead a successful intubation with a team.* |
| **Primary Learning Objectives** | 1. *Demonstrate the ability to organize and lead the care team for a successful intubation* 2. *Explain appropriate use of a bag valve mask* 3. *Formulate back up options for airway cases including bougie assisted intubation* |
| **Critical Actions** | 1. *Promptly recognize critical illness*    1. *Verbalize acuity, and recruit multiple healthcare workers to assist*    2. *Obtain intravenous (IV) access, place on monitor, apply oxygen, and obtain vital signs* 2. *Recognize need for intubation*    1. *Verbalize failure to protect airway, and respiratory failure* 3. *Recognize need for backups for possible difficult intubation*    1. *Verbalize if anticipated difficult airway*    2. *Collect adjuncts and difficult airway equipment (Naso-pharyngeal airway (NPA), oral-pharyngeal airway (OPA), stylet, bougie, scalpel/cricothyrotomy kit)* 4. *Promptly lead team in successful intubation*    1. *Attempt to pre-oxygenate with non-rebreather or nasal cannula at high flow rate*       1. *Suction out airway secretions*       2. *Provide bag-valve-mask (BVM) ventilations*          1. *Achieve mask seal with minimal leak*          2. *Avoid compressing airway, lift face into mask*          3. *Provide measured administration of breaths (over 1 second)*          4. *Avoid hyper (>20 breaths/min) or hypo (<12 breaths/min) ventilation*       3. *Use adjuncts as necessary (NPA, OPA) to assist in pre-oxygenation*    2. *Assemble equipment, at minimum:*        1. *BVM attached to O2, suction, laryngoscope (confirmed functional), endotracheal tube (size 7.0-8.0, confirmed intact balloon), stethoscope*    3. *Recognize and utilize an acceptable sedative at correct dose for RSI:*       1. *Eg Etomidate, 0.2-0.4mg/kg IV, Ketamine, 1-2mg/kg IV, or Propofol 1-3mg/kg IV*    4. *Perform endotracheal intubation, and verbalize confirmation with multiple methods (eg direct visualization, fog in tube, absent epigastric sounds, bilateral chest rise, bilateral breath sounds)*        1. *If difficulty, utilize bougie for placement of tube*       2. *Avoid dental trauma by minimizing contact with teeth and avoiding using teeth as fulcrum for laryngoscope tilting*    5. *Ensure (if cuffed tube) that cuff is inflated to appropriate level (5-10ml)*    6. *Ensure tube not displaced*       1. *Do not release ETT until securely fixed with tape or securement device*    7. *Secure at appropriate initial depth (e.g. ETT size x 3)* 5. *Discuss appropriate post-intubation care*    1. *Provide appropriate post-intubation sedation*       1. *E.g. benzodiazepine or propofol drips, plus fentanyl drip*    2. *Obtain and interpret STAT post-procedural CXR to confirm tube position*    3. *Order arterial blood gas* |
| **Learner Preparation** | *Prior to the case, learners are taught how to run an intubation. They are taught to use the mnemonic SOAP ME (Suction, Oxygen, Airways, Positioning, Medications, Equipment) to help remember how to prepare for an intubation.*   1. Suction: suction turned on 2. Oxygen: Bag valve mask ready, Nasal cannula on patient with 15L O2 and Non-rebreather mask on patient 3. Airways: Oral and nasal airways. ETT tube (discuss different sizes for adults) 4. Positioning: Ear to sternal notch position. Upright positioning as alternative. 5. Medications: Discuss different RSI medications and doses:    1. Induction agents:       1. Ketamine, Etomidate, Midazolam, Propofol       2. Succinylcholine, Rocuronium    2. Post intubation sedation medications:       1. Propofol, fentanyl, midazolam 6. Equipment: laryngoscope (curved or straight blade and sizes), bougie as back-up. (No video in actual emergency room or end-tidal CO2)   They are also taught techniques for intubation including   1. Personal protective equipment 2. SOAP ME 3. Grasp laryngoscope in left hand 4. Open patient’s mouth with cross finger technique with R hand 5. Slowly insert blade into right side of patient’s mouth and use it to sweep the tongue to the left. Advance the blade inward. 6. If using a curved blade- the tip should be placed in the vallecula. If using a straight blade, the tip should be used to pull the epiglottis out of the way 7. Once blade in correct position, left handle without applying backward pressure toward patient’s head 8. Grasp ETT tube with stylet with R hand and insert under direct visualization of vocal cords. ETT tube depth should be around 3x ETT size at the teeth.    1. If cannot visualize the entire vocal cords, can use a bougie.    2. Describe Seldinger technique with either a pre-loaded bougie or an assistant placing the ETT over bougie when requested.    3. Describe using the coude tip to help with anterior airways 9. Hold ETT in place and withdraw the blade. 10. Remove the style and inflate the ETT cuff with 5-10 ml of air 11. Confirm tube placement (listen to gastric sounds, listen to breath sounds, condensation in tube, look at pulse oximeter, symmetric chest movement. 12. Secure ETT with tape 13. Instruct learners that ventilation is more important the intubation. If unable to intubate, go back and ventilate the patient and think of using back ups or calling for help. |

| Initial Presentation | | | |
| --- | --- | --- | --- |
| **Initial vital signs** | T 37, BP 120/80, Sat on room air 90%, RR 10, HR 80 | | |
| **Overall Appearance** | *Unresponsive patient.* | | |
| **Actors and roles in the room at case start** | *There is nurse available to help.* | | |
| **HPI** | *Patient is a 26 year old on the medical floor who is unresponsive. The medical team is requesting the provider to intubate the patient.* | | |
| **Past Medical/Surgical History** | **Medications** | **Allergies** | **Family History** |
| None | None | None | Non-contributory |
| **Physical Examination** | | | |
| **General** | Unresponsive | | |
| **HEENT** | Atraumatic. Pupils equal and reactive. No trismus | | |
| **Neck** | Supple | | |
| **Lungs** | Clear bilaterally | | |
| **Cardiovascular** | Normal sinus rhythm | | |
| **Abdomen** | Soft, non-distended | | |
| **Neurological** | Unresponsive | | |
| **Skin** | Normal | | |
| **GU** | Normal | | |
| **Psychiatric** | Unresponsive | | |

| Instructor Notes - Changes and CASE Branch Points | | |
| --- | --- | --- |
| **Intervention / Time point** | **Change in Case** | **Additional Information** |
| If learners do to verbalize preparation of intubation |  | RN will ask for direction |
| Learners call for medications |  | RN will ask for dosages if not requested. |
| After intubation |  | RN will ask for sedation medications if not requested |

**Ideal Scenario Flow**

*The learners enter the room and recognize need for intubation. They ask the nurse to obtain IV access and place patient on monitor. They ask for the suction to be prepared, pre-oxygenate the patient with nasal cannula and face mask. They ask for the equipment (laryngoscope blade, endotracheal tube size) and medications with appropriate dosing. They position the patient optimally, ask for bougie as backup, and direct the team for successful intubation. After intubation, they ask for appropriate sedation medications.*

**Anticipated Management Mistakes**

1. *Lifting the laryngoscope blade up and away instead of rotating backwards: our learners often used the teeth as a fulcrum. We were able to perform active feedback as learners practiced the procedure component of the intubation by teaching optimal control of the blade.*
2. *Vocalizing each step and directing team members*: *our learners would forget steps of setting up or would not verbalize each step. During debriefing we would go over the mnemonic SOAP ME for a basic airway set up and discussed the importance of using the entire team for assistance.*
